# Thiamine pyrophosphokinase deficiency: report of two Chinese cases and a literature review

**DOI:** 10.3389/fped.2023.1173787

**Published:** 2023-08-09

**Authors:** Dan Zhao, Ming Liu, Huafang Jiang, Tianyu Song, Chaolong Xu, Xin Duan, Ruoyu Duan, Han Xu, Zhimei Liu, Fang Fang

**Affiliations:** Department of Neurology, National Center for Children’s Health, Beijing Children’s Hospital, Capital Medical University, Beijing, China

**Keywords:** *TPK1*, TPK deficiency, thiamine pyrophosphokinase deficiency, outcome predictors, literature review

## Abstract

Thiamine pyrophosphokinase (TPK) deficiency, is a rare autosomal recessive disorder of congenital metabolic dysfunction caused by variants in the *TPK1* gene. *TPK1* variants can lead to thiamine metabolic pathway obstacles, and its clinical manifestations are highly variable. We describe two cases of TPK deficiency with completely different phenotypes and different therapeutic effects, and 26 cases of previously reported were retrospectively reviewed to improve our understanding of the clinical and genetic features of the disease. Patients with TPK deficiency present with ataxia, dysarthria, dystonia, disturbance of consciousness, seizures, and other nervous system dysfunction. Different gene variant sites may lead to different clinical features and therapeutic effects. Gene analysis is important for the diagnosis of TPK deficiency caused by *TPK1* variants, and thiamine supplementation has been the mainstay of treatment for TPK deficiency to date.

## 1. Introduction

**Figure 1:**
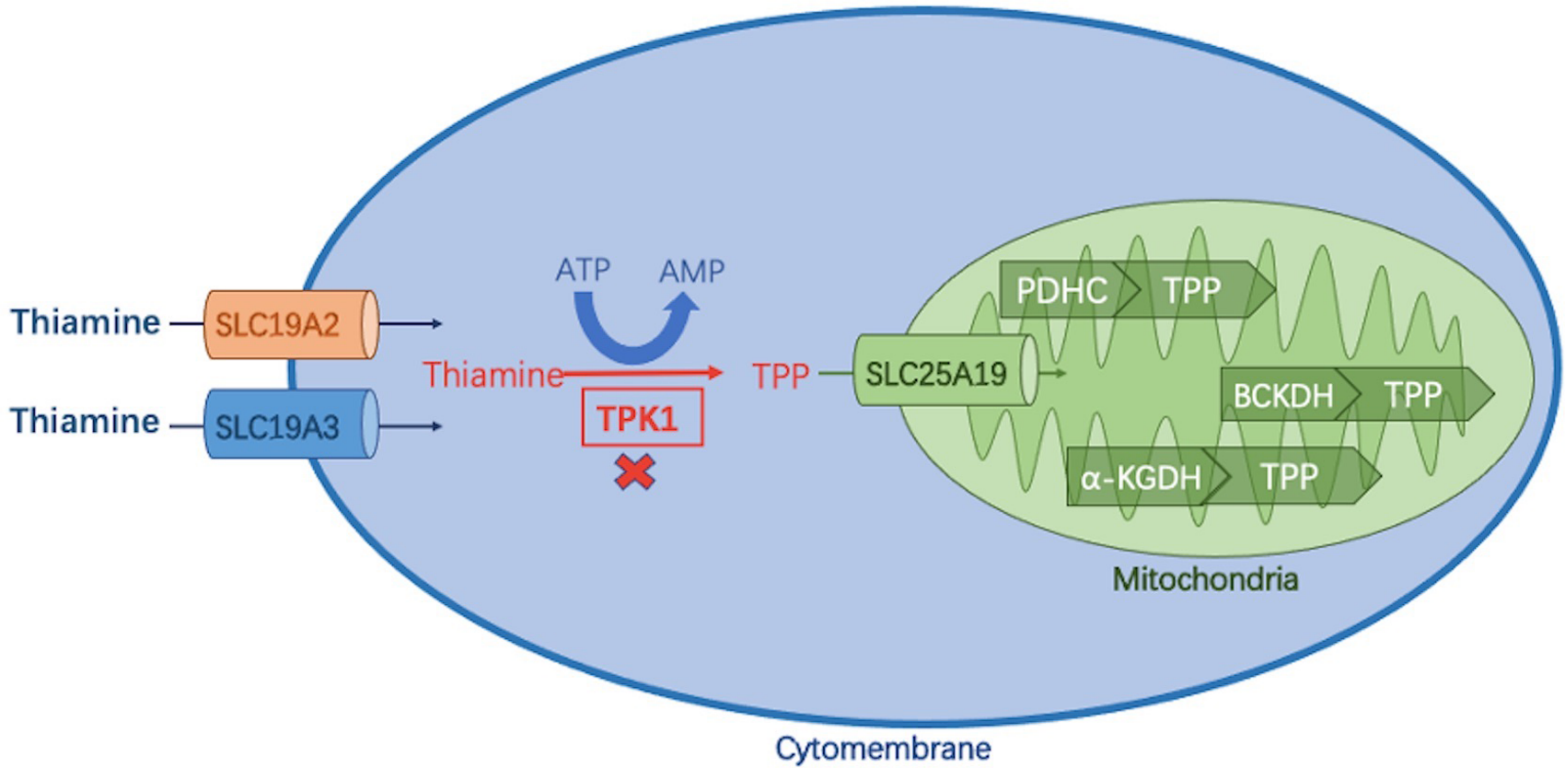
Thiamine metabolic pathway. Thiamine is absorbed in the small intestine and enters cells via two transporters (encoded by SLC19A2/SLC19A3), where it is phosphorylated to thiamine pyrophosphate (TPP) by thiamine pyrophosphate kinase 1 (TPK1). TPP enters the mitochondria via the SLC25A19-encoded carrier and acts as a cofactor of three different dehydrogenases to complete the energy metabolic pathway: (1) pyruvate dehydrogenase complex (PDHC); (2) branched α-ketoate dehydrogenase (BCKDH); α -ketoglutarate dehydrogenase (α-KGDH).

Thiamine (or vitamin B1) and its phosphorylate derivatives, thiamine monophosphate (TMP), thiamine pyrophosphate diphosphate (TPP), and thiamine triphosphate (TTP), are involved in energy metabolism and synthesis of nucleic acids, antioxidants, lipids, and neurotransmitters (1, 2). Different derivatives and metabolic pathways of thiamine are shown in Figure 1.

Four genes, *SLC19A3* (OMIM#606152), *SLC25A19* (OMIM#606521), *SLC19A2* (OMIM # 603941), and *TPK1* (OMIM#614458), are associated with thiamine metabolic disorders. Three of the genes *SLC19A3*, *TPK1*, and *SLC25A19*, are associated with acute encephalopathy, basal ganglia disease, and lactic acid accumulation caused by brain energy failure (3). Thiamine Metabolism Syndrome 5 (OMIM#614458) caused by *TPK1* gene variants is a congenital thiamine metabolic disorder with a highly variable phenotype. The human TPK1 gene (hTPK1) is located on 7q35 with a full-length cDNA of 2,439 bp and encodes a protein composed of 243 amino acids. It contains nine exons and encodes proteins starting from the second exon. Thiamine pyrophosphate kinase 1 encoded by the TPK1 gene plays an important role as a thiamine activator in cells. TPK protein functions as a dimer, containing only one domain, and further enters mitochondria to play a role in oxidative phosphorylation of pyro phosphorylating thiamine into thiamine pyrophosphate (TPP) (4).TPP is an active form of thiamine and a key cofactor of several enzymes that play major roles in energy metabolism (5, 6). Compared with patients with *SLC19A3* and *SLC25A19*, patients with *TPK1* variants presented with symptoms earlier, responded differently to thiamine supplementation, and developed a more severe phenotype with higher rates of morbidity and mortality (7). According to previous reports, not all patients with *TPK1* defects responded to thiamine therapy. The two previously unreported *TPK1* variants in this study elicited distinct responses to thiamine supplementation, which may be related to their age at onset, phenotype, and early start of high doses of parenteral thiamine treatment. Here, we describe two cases of TPK deficiency with completely different phenotypes and different therapeutic effects. Additionally, through literature search and analysis, 26 cases of previously reported patients were retrospectively analyzed to improve our understanding of the clinical and genetic features of the disease.

## 2. Patients and methods

### 2.1. Patient selection

In this study, two patients with *TPK1* deficiency were recruited from the Department of Neurology of Beijing Children's Hospital. Both of them had significant neurological symptoms and were diagnosed by whole exon sequencing (WES).

### 2.2. Literature review

**Figure 2:**
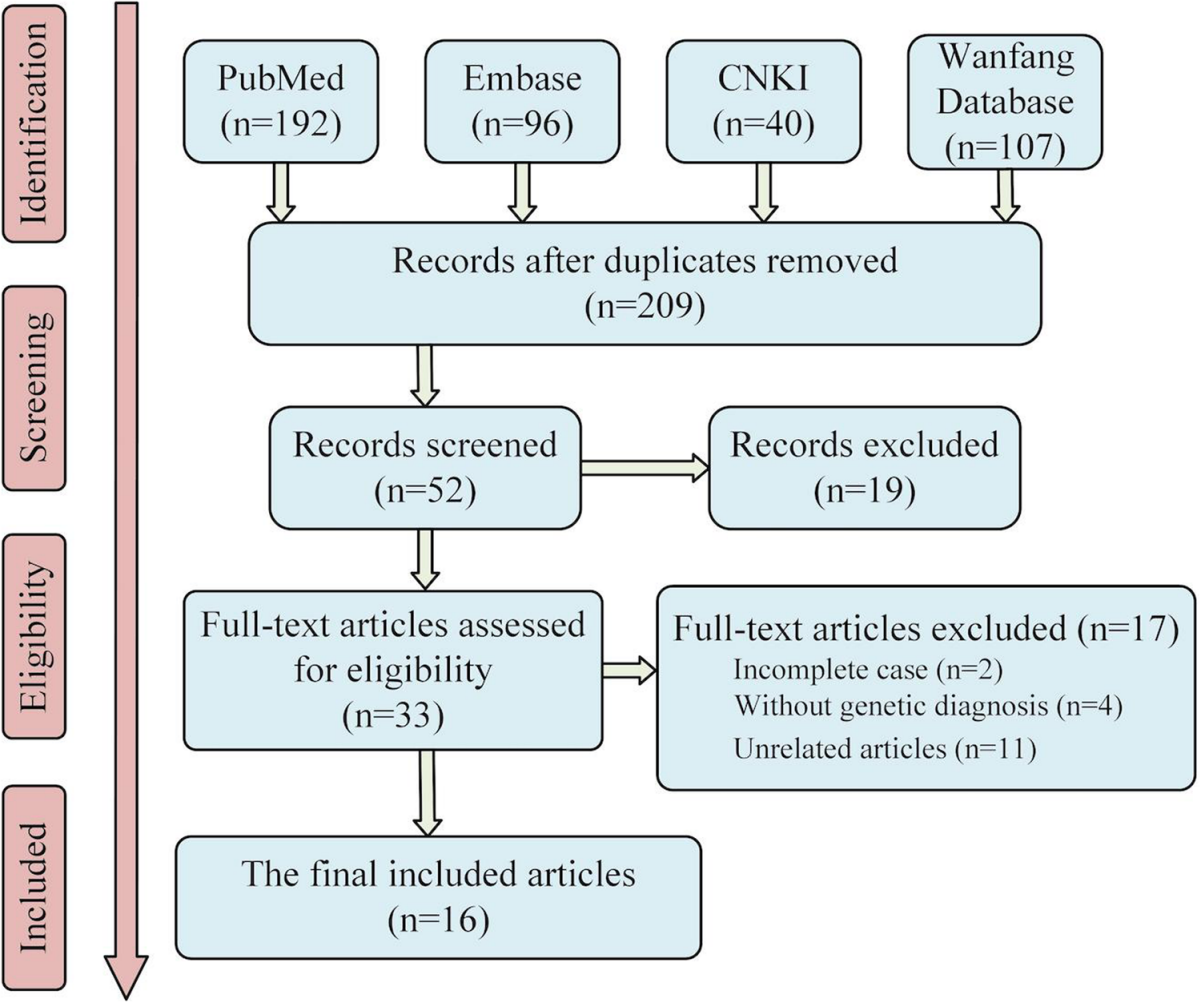
Literature screening flow chart. Sixteen articles were finally included in the analysis. Considering the two cases reported in this paper, twenty-eight patients with *TPK1* gene defect were included in this study.

The keywords, “*TPK1*”, “TPK deficiency”, “thiamine pyrophosphatase deficiency”, and “Thiamine pyrophosphatase deficiency”, were used as search terms in Embase, PubMed database, CNKI database, and Wanfang Database for literature review. A total of 209 articles were retrieved after removing duplicate items, which were retrieved from the database built up to February 2022. After excluding articles with incomplete cases, articles without genetic diagnoses, and irrelevant articles, 17 articles comprising 26 cases (5–20) were included (screening process is shown in Figure 2). Including the two cases reported in this study, we have shown the phenotype, genotype, clinical information, and treatment of 28 patients in the Supplementary Material.

### 2.3. Statistical analyses

All patients reported in the literatures were classified into Leigh group and non-Leigh group based on whether they had bilateral symmetric basal ganglia or cerebellar lesions on brain MRI. All patients treated with thiamine were divided into effective group (obvious improvement and certain improvement) and ineffective group (no improvement and death) according to their therapeutic efficacy.

Continuous variables were summarized with standard descriptive measures, including means, standard deviation, median, and interquartile ranges, whereas categorical variables were summarized using frequencies and percentages. For continuous variables, differences among comparison groups were estimated using the student *t*-test for normally distributed variables and the Wilcoxon rank-sum test for variables that were not normally distributed. Differences in categorical variables were assessed using the Chi-square test or the Fisher's Exact test. A *p*-value of <0.05 was considered significant. All statistical analyses were performed using IBM SPSS Statistics 22 software (IBM Corp., Armonk, NY).

## 3. Results

### 3.1. Patients

#### 3.1.1. Case 1

A 1-year and 8 months old boy first presented with unsteady gait and dysarthria of a week's duration. Before the onset, the patient had a fever of 39°C, rashes, and a convulsive seizure. Afterwards, unsteady gait and dysarthria, appeared and progressively worsened. The patient had no other infectious symptoms, such as cough, vomiting, and diarrhea. He had no hearing loss, intellectual disability, or other symptoms. He was the second child of a nonconsanguineous family and uneventfully delivered naturally at full term. His birth weight was 3 kg, and he had a suspicious history of intrauterine hypoxia at birth. Developmental milestones were normal before onset.

Physical examination showed unsteady walking and sitting, wide-based gait, dystonia, and dysarthria. Other cranial nerve examinations were unremarkable. Bilateral tendon reflexes were symmetrical; muscle strength and tone were normal; and pyramidal signs and meningeal irritation signs were negative.

**Figure 3:**
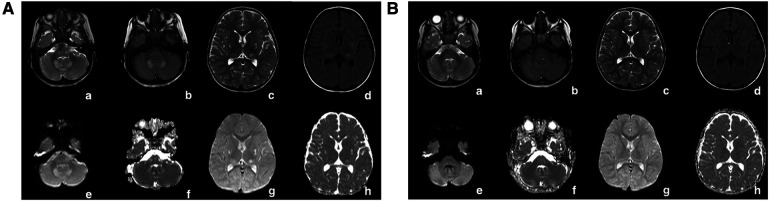
(**A**) In patient 1, at the onset of the disease (1 year and 8 months old), the cerebral MRI showed symmetrical patchy long T2 signals in the cerebellar dentate nucleus, bilateral basal ganglia, and thalamus. (**B**) In patient 1, the signal of long T2 in the brain improved gradually after vitamin B1 treatment for 1 year. The long patchy T2 signals were significantly attenuated on T2WI (a,e), T2-FLAIR (b,c), and DWI (c,g) sequences.

Blood lactic acid, blood ammonia, and homocysteine levels were normal. Urine screening showed mild keto-dicarboxylic acid urine with slightly higher levels of several organic acids. Blood screening showed no obvious abnormalities. Brain MRI showed symmetrical patchy long T2 signals in the cerebellar dentate nucleus, bilateral basal ganglia, and thalamus (Figure 3A). Gesell Developmental Assessment was performed, and cognitive area and personal-social area were normal. The patient had a mild developmental delay in the adaptation area, gross motor area, and fine motor area. A mitochondrial genetic test revealed a negative finding.

**Figure 4:**
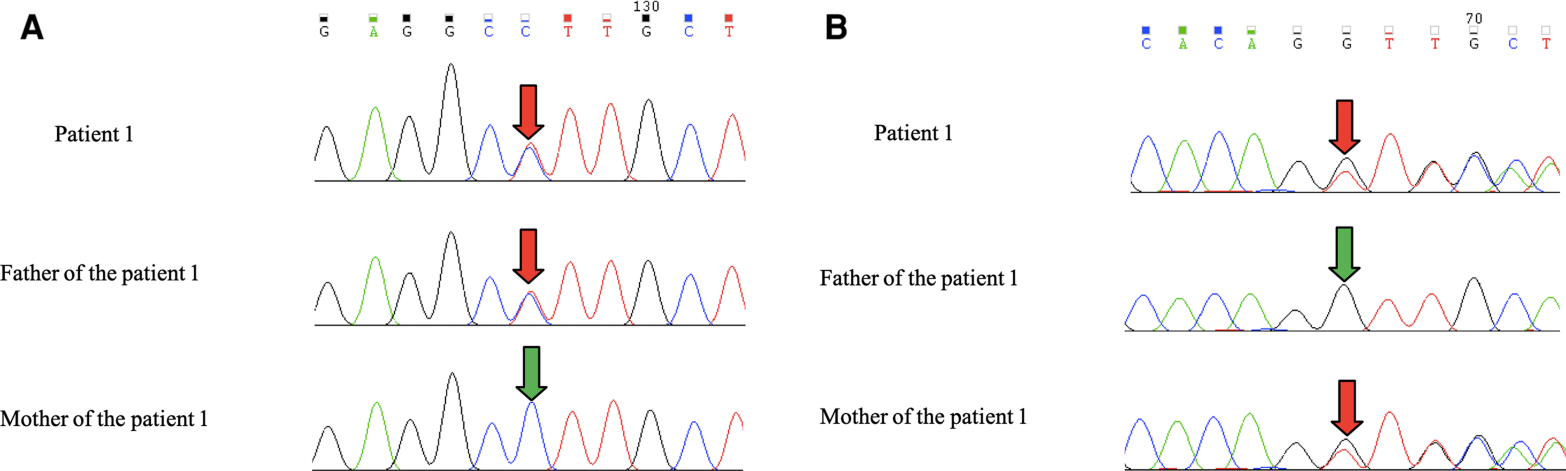
(**A**,**B**) Patient 1 had the c.513delG + c.382C>T complex heterozygous variant. The father carried the *TPK1* gene c.382C>T heterozygous variant, and the mother carried the *TPK1* gene c. 513delG heterozygous variant.

Leigh Syndrome was diagnosed and treated with cocktail therapy (thiamine 100 mg/day, riboflavin 100 mg/day, vitamin C 50 mg/day, vitamin E 100 mg/day, and coenzyme Q10 90 mg/day). After 2 months of treatment, his symptoms improved and he was followed up in the outpatient department for a long period. After 1 year of treatment, multiple abnormal signals previously observed in the brain MRI had reduced (Figure 3B). At the age of 4 years, symptoms recurred with ataxia, dysarthria, and inability to walk unaided after discontinuation of the drug. The symptoms gradually resolved after repeating the drug regimen (thiamine 100–200 mg/day oral treatment). Whole exon sequencing (WES) was performed after the parents agreed and signed informed consent. Compound heterozygous variants of *TPK1* in the patient (NM_022445.3), c.513delG (p.Arg171Ser fs*3) and c.382C>T (p.Leu128Phe), were identified (Figures 4A,B). The WES results supported the diagnosis of *TPK1*-associated Leigh syndrome. Oral treatment with a large dose of thiamine (10 mg/kg·day) was continued with the discontinuation of other medications.

The patient was followed up regularly annually. In the past 2 years, due to the COVID-19 pandemic, in-person follow-up was replaced by online video consultations. At the age of 6 years and 7 months, he discontinued against medical advice for 1 month, leading to a recurrence of the symptoms of walking instability and dysarthria. The symptoms disappeared after 2–3 days of remedication (200 mg/day). At the last follow-up, the patient was 9 years and 2 months old. He took thiamine regularly and had a normal life without ataxia and other symptoms.

#### 3.1.2. Case 2

An 8 months old male infant first reported to the hospital with developmental delay after birth and convulsions from the age of 1 month. The patient had no definite infectious history before the onset. At the age of 1 month old, seizures were tonic seizures, which lasted about 1–2 min, and occurred 4–5 times per month. At the age of 4 months, seizures became spasms, which were 30–40 clusters per event, and occurred once or twice per week. Developmental milestones were delayed after birth. He could not recognize and move towards the direction of sound and objects, raise his head, turn over, or sit. He was the second child of his parents, had a healthy sister, and his mother had an abortion. Family and birth histories were normal.

Physical examination revealed a microcephaly, inability to chase sound and objects, occasional nystagmus, and dystonia with hands clenched and thumbs adducted, presence of symmetrical bilateral patellar tendon reflexes, negative meningeal irritation sign, limb muscle power of 4, and hypertonia.

**Figure 5:**
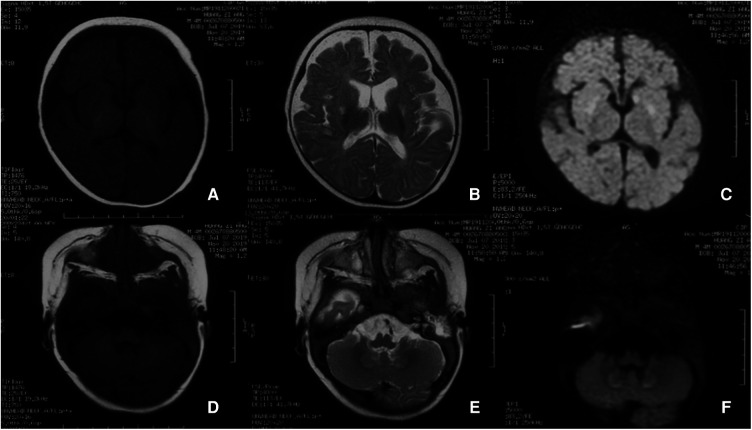
Brain MRI of case 2 at the beginning of the disease. Presence of roughly symmetrical abnormal signal foci in the head of the caudate nuclei, lenticular nuclei, and dentate nuclei of the cerebellar hemisphere.

Metabolic analysis of blood showed a lactate level of 3.8 mmol/L and high level of pyruvate (0.2 mmol/L). Organic acid in urine analysis showed significantly increased levels of urine 2-ketoglutarate and several organic acids. The activity of mitochondrial respiratory chain enzyme complexes was normal. At the age of 5 months, a muscle biopsy was performed and muscle pathology showed slight pathological changes. These changes included presence of hypertrophic, atrophic, and necrotic myofibers; increased poisoning of fat droplets in myofibers, and increased number of MHC-1-positive muscle fibers. The electroencephalogram showed an atypical hypsarrhythmia, and tonic seizures and clusters of spasms were observed. Brain MRI showed abnormal signals in the head of the caudate nuclei, lenticular nuclei, and dentate nuclei of the cerebellar hemisphere (Figure 5). WES was performed after the parents agreed and signed informed consent. Compound heterozygous variants of *TPK1*, (NM_022445.3) c. 263G>A (p.Cys88Tyr) and c.465_467delAAT (p. Leu156del), were identified.

Several antiepileptic drugs, such as topiramate, levetiracetam, and vigabatrin, were used to control the seizures but were less effective. The patient underwent concurrent cocktail therapy, including oral administration of thiamine (15–20 mg/kg·day), L-carnitine, biotin, coenzyme Q10, vitamin C, and vitamin E. At the age of 10 months, the patient underwent another brain MRI, which showed that the initial lesion had disappeared, the brain had atrophied, and the ventricles had enlarged. At the age of 1 year, although receiving daily doses of thiamine 100 mg intramuscularly and 100 mg orally, the patient could not chase sound and objects and raised his head unstably. More than 10 episodes of seizures were observed per day.

At the age of 1 year and 1-month-old, the patient was fed with a ketogenic diet (ratio 3:1), which reduced frequency of seizures. However, the treatment was discontinued at the age of 1 year and 7 months due to intolerance to the ketogenic diet. The patient died of encephalopathy and respiratory failure at the age of 2 years and 3 months.

## 4. Literature review

### 4.1. Demography

**Figure 6:**
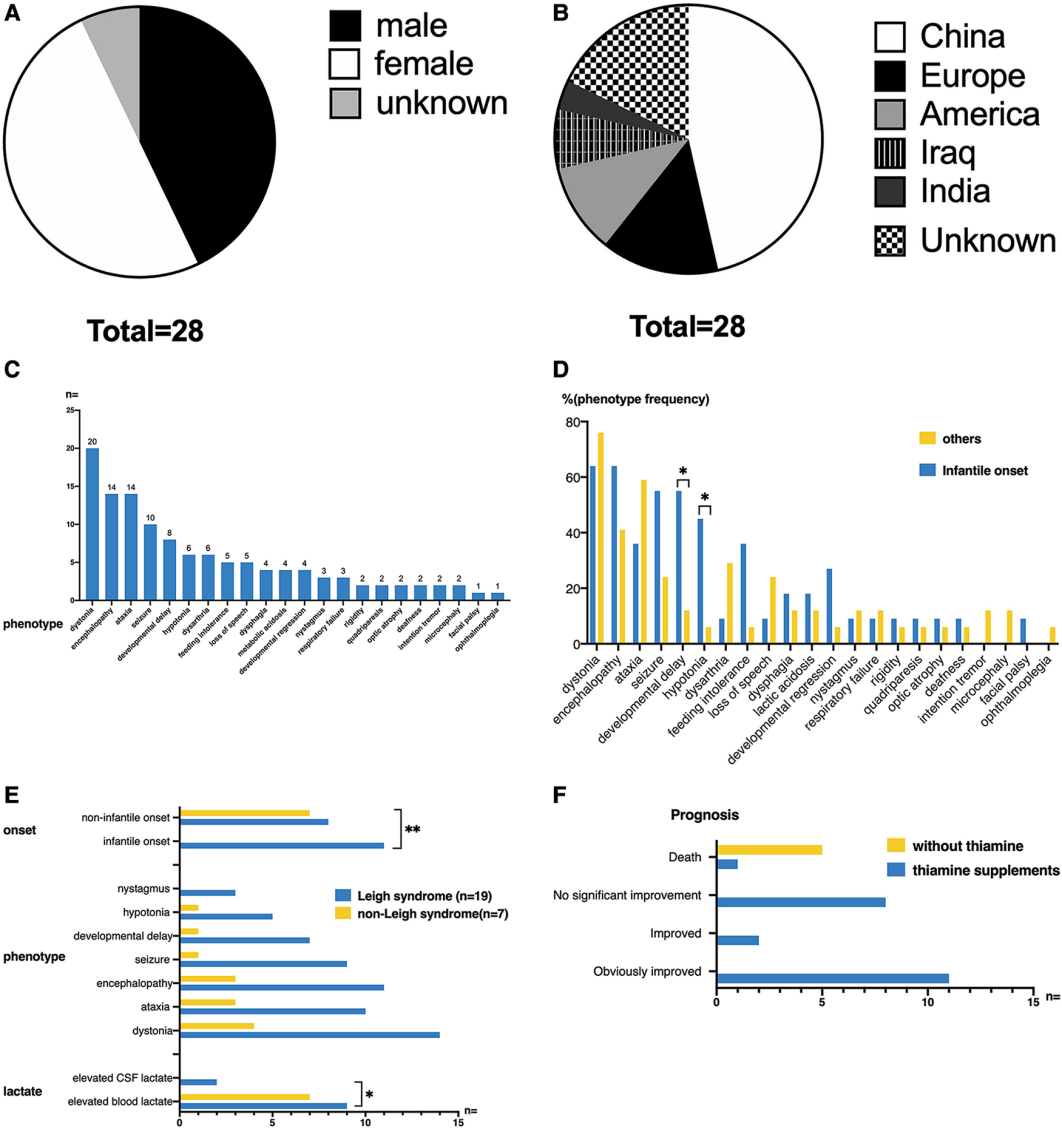
(**A**,**B**) Gender distribution and ethnic origin of all published cases of *TPK1* gene defect. (**C**) The clinical phenotypes of twenty-eight patients. The most common clinical manifestations included dystonia, ataxia, encephalopathy, seizure, and developmental delay. The most common signs included hypotonia and nystagmus. (**D**) Phenotypic differences between infantile-onset and non-infantile-onset patients. Among all phenotypes, developmental delay and hypotonia were more common in patients with infantile-onset (Fisher's Exact Test, *p* < 0.05). (**E**) Differences in clinical features between patients with Leigh syndrome and non-Leigh syndrome. The onset age of patients with Leigh syndrome was more distributed in the infantile onset group (Fisher's Exact Test, *p* = 0.01). Elevated blood lactate level was more common in the non-Leigh syndrome group (Fisher's Exact Test, *p* < 0.05). (**F**) Thiamine supplementation in patients with *TPK1* gene variants and related outcomes. Among the twenty-eight patients, twenty-two patients received thiamine supplementation (20–750 mg/day), eleven patients had significant improvement in clinical symptoms, two patients had partial improvement, and nine patients had no significant improvement [one of these nine patients (case 2 of this study) died].

The 28 patients included twelve (42.86%) males, fourteen (50%) females, and two (7.14%) unknown genders. Among them, nine (32.14%) patients had consanguineous parents. In terms of country and area distribution, thirteen (46.43%) patients were from China, five (14.29%) patients were from Europe (two cases from Germany, one case from Finland, and one case from Switzerland), three (10.71%) patients from the United States, two (7.14%) patients from Iraq, and one (3.57%) patient from India. The nationalities of five (17.86%) patients were unknown (Figures 6A,B).

### 4.2. Symptoms and signs

For these patients, the onset of disease was mostly during infancy (11 cases, 39.29%), early childhood (11 cases, 39.29%), early school age (5 cases, 17.85%), and unknown (1 case, 3.57%). Twenty (71.43%) cases were preceded by an infection or trauma. The clinical phenotypes of the 28 patients are shown in Figure 6C. The most common clinical manifestations were dystonia (20/28, 71.43%), ataxia (14/28, 50%), encephalopathy (14/28, 50%), seizure (10/28, 35.71%), and developmental delay (8/28, 28.57%). The most common signs included hypotonia (6/28, 21.43%) and nystagmus (3/28, 10.71%). Other symptoms and signs observed in few patients were rigidity (2/28, 7.14%), quadriparesis (2/28, 7.14%), optic atrophy (2/28, 7.14%), deafness (2/28, 7.14%), intention tremor (2/28, 7.14%), microcephaly (2/28, 7.14%), facial palsy (1/28, 3.57%), and ophthalmoplegia (1/28, 3.57%).

According to age of onset, patients were divided into an infantile-onset group and non-infantile onset group (as shown in Figure 6D). Among all phenotypes, developmental delay and hypotonia were more common in patients with infantile onset, and the differences were statistically significant (*p* < 0.05).

### 4.3. Lab examination and neuroimaging

Blood lactic acid tests performed in 26 cases revealed elevations in sixteen (16/26, 61.53%) cases (reference range: 0.5–1.7 mmol/L), and lactic acidosis during the course of the disease in two cases. Only two (2/14, 14.29%) patients had elevated levels of cerebrospinal fluid (reference range: 1.0–2.8 mmol/L). The levels of thiamine pyrophosphate (TPP) in blood and muscle were measured in some patients. Muscular concentrations of TPP were decreased in all five patients (5/5, 100%), and TPP concentrations in blood were decreased in eight patients (8/12, 66.67%). Organic acid in urine analysis was performed in nineteen patients; eleven patients (11/19, 57.89%) showed elevated urine 2-KGA levels, and six patients (6/19, 31.58%) had normal levels of urine 2-KGA. Pyruvate levels were measured in eight patients, that were elevated in six patients (75%). The skin/muscle respiratory chain complex activity was measured in nine patients (P2, P3, P5–P10, P16), which revealed slightly decreased (3/9, 33.33%) or normal activity (6/9, 66.67%). We observed a slight decrease in complex V enzyme activity in P2 muscles; complex I, complex II, complex III, and complex I + III in P3 muscles; and complex III in P5 muscles.

Brain MRIs were performed in 26 patients at the acute stage. Five patients had normal cerebral MRI findings, whereas twenty-one patients (80.77%) had abnormal brain MRI findings, which mainly involved the bilateral basal ganglia (50%), thalamus (23.08%), and especially cerebellum (50%). In few cases, the brainstem, corpus callosum, and spinal cord were involved. A diagnosis of Leigh syndrome was made in nineteen cases (73.08%). Brain MRI findings in seven cases (26.92%) showed that original lesions improved or disappeared after thiamine treatment.

According to the presentation of cerebral MRI, 26 children with acute state MRIs were divided into the Leigh syndrome group (19/26, 73.08%) and the non-Leigh syndrome group (7/26, 26.92%). As shown in Figure 6E, the onset age of patients with Leigh syndrome was highly distributed during infancy (*p* = 0.01). No significant differences in symptoms and signs were observed between the Leigh syndrome group and the non-Leigh syndrome group. Elevated blood lactate was more common in the non-Leigh syndrome group (*p* < 0.05).

### 4.4. Treatment and outcome

As shown in Figure 6F, twenty-two patients received thiamine supplementation (20–750 mg/day), five patients did not receive thiamine supplementation, and one patient did not mention treatment and prognosis. Among patients who received thiamine supplementation, eleven (11/22, 50%) patients had significant improvement in clinical symptoms, two (2/22, 9.09%) patients had partial improvement, nine (9/22, 4.91%) patients had no significant improvement, in which one patient (1/22, 4.55%) died. All five patients who did not receive thiamine supplementation died. The survival rate was higher in the thiamine-treated group (21/22, 95.5%) than in the thiamine-untreated group (0/6, 0.0%) (*p* < 0.01). Among the six dead patients, two patients had unknown causes, one patient died of viral infection at 3.5 years old, and three other patients with clinically severe recurrent encephalopathy and refractory epilepsy died of metabolic crisis, respiratory failure, and multi-organ failures. The mean age at death was 4.72 years, and the median age was 2.96 years (0.5–11 years).

**Figure 7:**
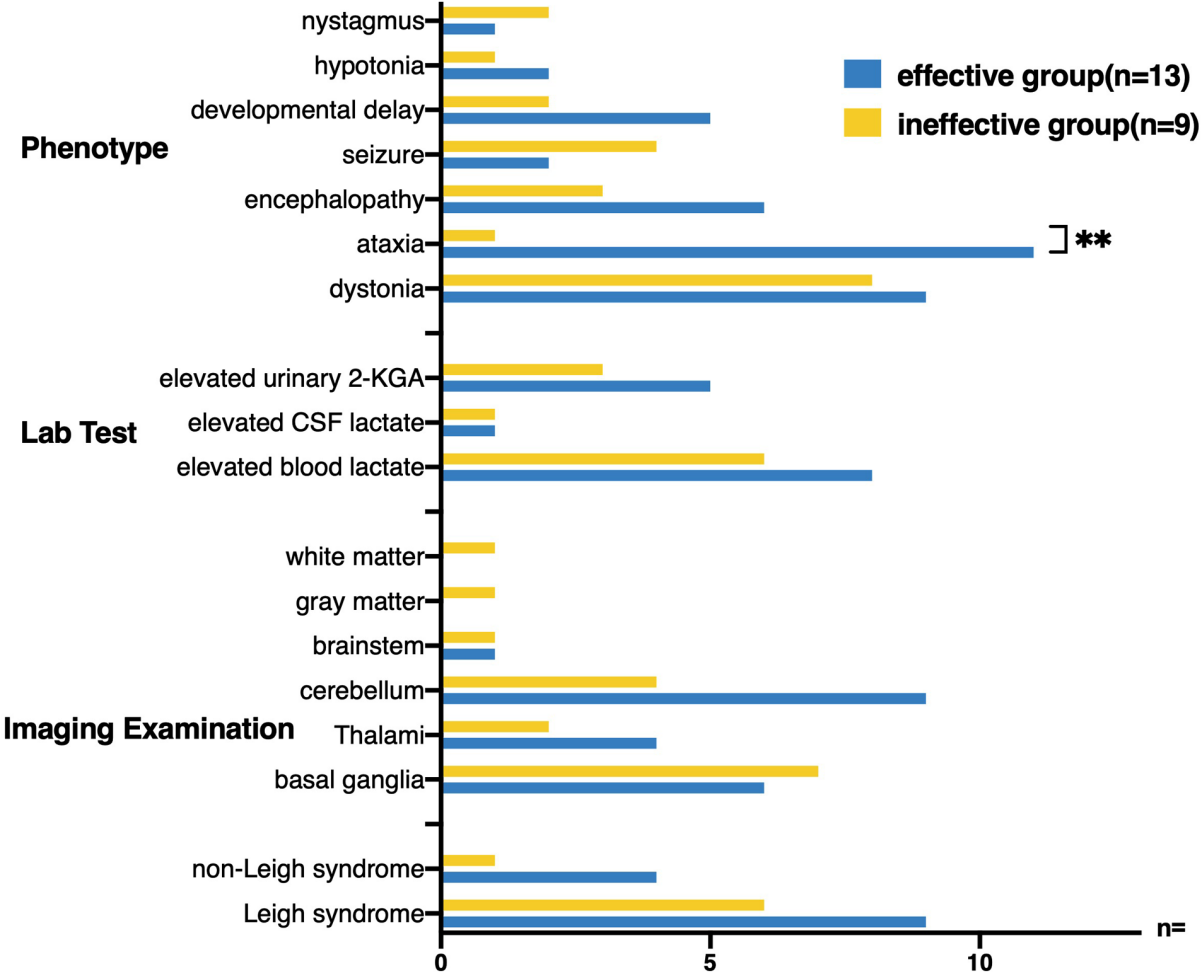
Twenty-two patients receiving thiamine treatment were divided into effective treatment group and ineffective treatment group, and the clinical characteristics of the two groups were compared. Ataxia onset was more common in the effective treatment group (84.62% VS 11.11%, Fisher’s exact test, *p* < 0.005), whereas seizures were more common in the ineffective treatment group (4/9, 44.44%). The cerebellum (9/13, 69.23%) was more likely to be involved in the brain MRI in the effective treatment group.

Twenty-two patients who received thiamine treatment were divided into the effective group (thirteen cases with significant clinical improvement or partial improvement) and the ineffective group (nine cases with clinical failure or death) (Figure 7). No difference in age of onset was observed between the two groups (1.64 ± 1.18 vs. 1.94 ± 1.83, *t*-test: *t* = –0.470, *p* > 0.1). The maximum reported age of survival was 40 years old. In terms of clinical symptoms, ataxia onset was more common in the effective group (84.62% vs. 11.11%, *p* < 0.005), whereas seizures were more common in the ineffective group (4/9, 44.44%). Although the basal ganglia involvement rate in the ineffective group (7/9, 77.78%) was higher than that in the effective group (6/13, 46.15%), no difference was observed (*p* > 0.05). The rate of increase in blood lactic acid level in the two groups was more than 60%; however, no significant difference was observed. Additionally, no significant difference in the proportion of patients with Leigh syndrome was observed between the effective group and ineffective group.

### 4.5. Genetic findings

**Figure 8:**
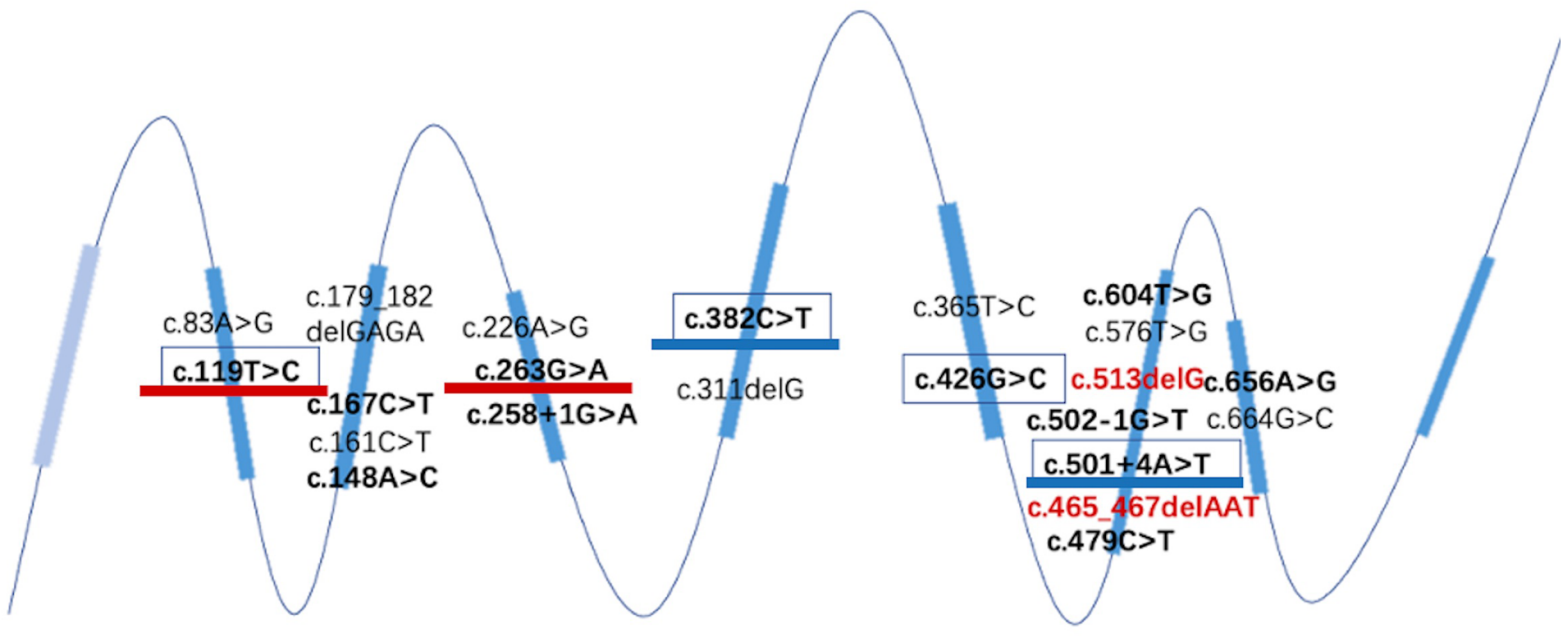
Structure of all reported *TPK1* gene variant loci. Red font represents the newly discovered variants in this study, all located in the seventh exon. The variants present in ≥3 cases have been highlighted. Effective therapeutic variants have been underlined in blue. Therapy-effective variants are underlined in blue. The sites of variants that did not respond to treatment are underlined in red.

The distribution of all discovered *TPK1* variants of the gene, including the four variants in this study, is shown in Figure 8. Twenty-two gene variants of the *TPK1* gene were found, and most of them were located in exon 7, followed by exon 3 and exon 2. Among the 22 gene variants, 15 missense variants, three splicing variants, three frameshift variants, and one fragment deletion variant were identified.

Of the twenty-eight patients, three had frameshift mutations confirmed by WES, and two of them received thiamine therapy, all of which were effective. There were twenty-five cases of non-frameshift mutation, of which sixteen were treated with thiamine and nine were effective (*p* > 0.05).

Among the twelve patients who underwent thiamine treatment, four patients had the c. 382C>T (P. Lys128Phe) variant and two patients had the C. 501 + 4A > T (p. Val119_Pro167del) variants (underlined in blue in Figure 6). Among the eight patients who did not respond to treatment, two and three patients had c.263G>A (p. Cys88Tyr) and C. 119T>C (p. lys40Pro), respectively (underlined in red in Figure 8). Patients who died had exon 3, 4, 6, or 7; and no aggregation of death cases was found in a specific exon or variant site.

## 5. Discussion

Herein, we report two cases of THMD5 with different clinical manifestations and different responses to thiamine treatment of *TPK1* variants. One patient presented with ataxia, and thiamine replacement therapy could achieve significant clinical efficacy. The other patient had refractory epilepsy, and thiamine combined with antiepileptic therapy had a poor prognosis.

### 5.1. The phenotype

Up to now, twenty-six patients from thirteen families, five of which are consanguineous, had been reported before the publication of this manuscript. The mean onset age of thiamine pyrophosphokinase deficiency caused by *TPK1* variants is 1.85 years. The cases of *TPK1* variants are most likely to occur in infants, but the mean time of diagnosis is approximately 5 years. Compared with other mitochondrial diseases, *TPK1* variants has various clinical phenotypes without specific clinical characteristics. In addition to the main symptoms, such as ataxia, encephalopathy, and dystonia, it can be combined with feeding intolerance and developmental delay or regression. Children with infantile onset of the disease are more likely to have developmental problems, whereas others are more likely to show ataxia. No significant difference in the age of onset of epilepsy and epileptic encephalopathy were observed.

Elevated lactate levels in the blood and cerebrospinal fluid were generally considered to be auxiliary diagnostic methods. A total of 61.5% of patients with TPK deficiency had elevated blood lactate level and only 21.4% of patients had elevated cerebrospinal fluid lactate level. Therefore, elevated blood/cerebrospinal fluid lactate seems not to be a specific biomarker of *TPK1* variants. In the patients who underwent the TPP concentration test, the blood/muscle TPP concentration decreased. Therefore, the decline in TPP concentration can be used as a specific biomarker for TPK defects, but it cannot be routinely tested in clinical practice due to the high level of requirements needed to establish for detection facilities. Organic acid in urine analysis revealed that urine 2-KGA level was elevated in more than half of the patients, which has certain suggestive significance for diagnosis. Since TPK defect affect pyruvate thiamine metabolism by leading to a lack of key enzyme cofactors, it can show a decrease in pyruvate level and PDC activity. However, the TPP does not affect the mitochondrial OXPHOS respiratory chain protein expression and activity, resulting in normal respiratory chain complex enzymes (10). This finding is consistent with findings from previously reported cases with high blood pyruvate level but normal respiratory chain enzyme activity.

A noteworthy finding was the presence of cerebellar involvement as the main imaging feature of the presence of *TPK1* variants. Based on imaging characteristics, patients were grouped into Leigh syndrome group and non-Leigh syndrome group. Patients in the Leigh syndrome group had a younger onset age and less elevated level of lactate in blood than the non-Leigh syndrome group. These findings are not often mentioned in previous literature. Whether this phenomenon is related to the difference in specific gene variation sites needs further detailed study.

TPK deficiency due to *TPK1* variants is a treatable disease. Some patients had mild clinical manifestations, especially those with ataxia. Thiamine supplementation immediately after early diagnosis or suspected diagnosis showed significant improvement or even normalization of clinical symptoms. However, failure to receive timely thiamine treatment can lead to irreversible brain damage and catastrophic consequences. In this study, death was recorded only among patients who were untreated. The only patient who received thiamine treatment but still had death as clinical outcome was the patient in case 2 in this study. This child had an early onset age and presented with severe epilepsy and encephalopathy clinically, but thiamine supplement therapy had no significant effect. Late treatment with a ketogenic diet was not well tolerated. TPK deficiency requires early recognition as one of the few treatable mitochondrial diseases. Suspected mitochondrial diseases, especially thiamine metabolic deficiencies, should be treated early with a thiamine cocktail in combination with other multivitamins. Thiamine should be dosed after genetic confirmation, but no standards for the recommended dose of thiamine are currently available. Most medical institutions use thiamine 100–400 mg/day (or 10 mg/kg/day). Larger long-term studies are needed to determine the appropriate dose of thiamine supplementation during and between onset. Additionally, Mayr et al. (8) proposed direct supplementation of TPP as the synthesis defect of TPP, which is a key component of the pathogenicity of *TPK1* variant. However, the effectiveness and safety of TPP have yet to be confirmed.

### 5.2. The genotype

In this study, two new variants of the TPK1 gene were reported; c. 513delG (p. Arg171SerFS *4) and c.465_467delAAT (p. Leu156del) were both frame-shift variants, which resulted in changes in the amino acid triad coding frame, thus affecting the structure and function of the coding protein. The ACMG ratings of the two variants were both potentially pathogenic. This site has never been reported by previous studies. Two cases of c. 382C>T (p. Leu128Phe) have been reported as a pair of twins who showed paroxysmal ataxia (c. 382C>T homozygous variant). L128F is a variant on a highly conserved residual, and L128 is located in mice's α/β domain of TPK crystal structure. It is related to the formation of TPK dimer and may destroy the function of the enzyme (21).

Few studies on the pathogenesis of *TPK1* variant have been reported. Banka et al. (9) reported that the enzyme activities of S160l and D222H mutants were lower than that of wild-type *TPK1*, but Huang et al. (13) found that the enzyme activity of most mutants was significantly reduced by testing the enzyme activity of various *TPK1* mutants, whereas the activities of S160l and W202G mutants were significantly higher than that of the wild-type. Furthermore, isothermal titration calorimetry was used to determine whether the variant affected the affinity of *TPK1* to thiamine. The affinity of S160l to thiamine was significantly reduced, and no bond between the W202G mutant and thiamine was detected. The decrease in bonds of S160l and W202G to TPP, a thiamine structurally similar product, increased enzyme activity. Additionally, Huang et al. (13) measured the melting temperatures (Tm) of the wild-type and mutant *TPK1* by differential scanning calmetry and found that the protein stabilities of four mutants (S160l, W202G, N219S, and D222H) were significantly lower than that of the wild-type. They suggest that the *TPK1* variant can alter thiamine metabolism by altering TPK1 enzyme activity, reducing the affinity of TPK1 for thiamine, or reducing the stability of *TPK1* protein (13). However, no cytological or animal studies have provided further evidence.

### 5.3. The relationship between phenotype and genotype

In the conclusion of a report on the prognosis of 22 thiamine-treated patients, no difference in onset age was observed between the effective treatment and ineffective treatment groups. More cases of ataxia was observed in the effective group, and brain MRI showed more cerebellar involvement. The variants, c. 382C>T (p. Lys128Phe) (17, 22) and c. 501 + 4A > T (P. Val119_Pro167del) (23, 24), were discovered more in the effective treatment group. c.263G > A (p. Cys88Tyr) (14) and C.119T > C (p. Lys40Pro) (8, 12) were more common in the ineffective treatment group. This finding is consistent with the finding of a difference in the therapeutic effect of the two cases in our study. Currently, few cases have been reported and variant sites are scattered, and no obvious correlation between genotype and clinical phenotype has been found. The specific mechanism needs to be further studied.

## 6. Conclusion

The clinical manifestations of THMD5 are highly variable. Therefore, the early diagnosis of TPK deficiency is a huge challenge. The clinical manifestations are acute onset encephalopathy or ataxia and unexplained seizures, especially with Leigh syndrome starting in infancy. Active and well-developed WES facilitates early molecular diagnosis. Meanwhile, for THMD5, a rare neurodegenerative disease, early supplementation with thiamine has a great effect on prognosis.

## Data Availability

The original contributions presented in the study are included in the article and supplementary table, further inquiries can be directed to the corresponding authors.
